# Host Directed Therapy for Chronic Tuberculosis via Intrapulmonary Delivery of Aerosolized Peptide Inhibitors Targeting the IL-10-STAT3 Pathway

**DOI:** 10.1038/s41598-018-35023-0

**Published:** 2018-11-09

**Authors:** Rashmi Upadhyay, Andrea Sanchez-Hidalgo, Carol J. Wilusz, Anne J. Lenaerts, Jennifer Arab, Joanna Yeh, Karen Stefanisko, Nadya I. Tarasova, Mercedes Gonzalez-Juarrero

**Affiliations:** 1 Department of Microbiology, Immunology and Pathology, Colorado State University, Fort Collins, CO 80523 USA; 2 Cancer and Inflammation Program, Center for Cancer Research, National Cancer Institute, Frederick, MD 21702 USA

## Abstract

Here we demonstrate that aerosols of host directed therapies [HDT] administered during a chronic *Mycobacterium tuberculosis* (Mtb) infection have bactericidal effect. The pulmonary bacterial load of C57BL/6 mice chronically infected with Mtb was reduced by 1.7 and 0.6 log_10_CFU after two weeks of treatment via aerosol delivery with ST3-H2A2, [a selective peptide inhibitor of the STAT3 N-terminal domain] or IL10R1-7 [selective peptide inhibitor for the IL-10Ra] respectively and when compared to control mice treated with IL10R1-14 [peptide inhibitor used as negative control] or untreated mice infected with Mtb. Accordingly, when compared to control mice, the bactericidal capacity in mice was enhanced upon treatment with peptide inhibitors ST3-H2A2 and IL10R1-7 as evidenced by higher pulmonary activities of nitric oxide synthase, NADPH oxidase and lysozyme enzymes and decreased arginase enzyme activity. This therapy also modulated important checkpoints [Bcl2, Beclin-1, Atg 5, bax] in the apoptosis-autophagy pathways. Thus, even in the absence of antibiotics, targeting of the host pulmonary IL-10-STAT3 pathway can significantly reduce the Mtb bacilli load in the lungs, modulate the host own bactericidal capacity and apoptosis and autophagy pathways. Our approach here also allows targeting checkpoints of the lungs to determine their specific contribution in pulmonary immunity or pathogenesis.

## Introduction

Current chemotherapy for tuberculosis (TB) fails to adequately control the global spread of *Mycobacterium tuberculosis* (Mtb) infection, the causative agent of TB. Treatment for drug-susceptible TB today requires 6–9 months of multidrug therapy^1^ whereas TB patients with multi-drug resistant (MDR)-Mtb infections endure two years of treatment that is unsuccessful in more than 50% of cases. To improve TB control globally, the World Health Organization is asking for short, effective and well-tolerated therapies for latent TB infection^2^. Such therapies will increase compliance and reduce the development of strains that are resistant to the new chemical entities^1^. One approach towards this goal is to combine host-directed therapies (HDT) with current therapies for TB.

Persistent exposure to the cytokine IL-10 inhibits several functions in macrophages and T cells including Th1 immune responses, apoptosis and autophagy^3–6^. IL-10 mediates all its functions via activation of STAT3 (signal transducer and activator of transcription 3), which in turn promotes cell proliferation and resistance to apoptosis^7–10^. Previous research in our lab and others has shown that Th1 responses against the Mtb bacilli are diminished in the presence of IL-10^11–18^. During Mtb infection, IL-10 activates STAT3 signaling in infected and surrounding cells, which in turn inhibits the bactericidal capacity of the macrophages by inducing production of Arginase 1 and inhibition of nitric oxide synthase (NOS)^15,19,20^. Moreover, STAT3 signaling in myeloid cells promotes Mtb infection^21–24^ and impairs T cell function in murine TB models^22^ and TB patients^21^. Thus, the goal of these studies was to determine if interfering with the pulmonary IL-10-STAT3 pathway can increase the bactericidal capacity of the host in the chronically *Mtb* infected lungs and can be used as a host targeted therapeutic approach.

Metabolically stable peptides targeting protein-protein interactions are increasingly used as drugs and drug leads^25^. Rational drug design has led to the development of potent and selective lipopeptide inhibitors of STAT3 N-terminal domain (ND)^25^ and IL-10 receptor alpha chain (IL10Ra). The peptide inhibitor of STAT3 ND (ST3-H2A2) was developed to impair persistent activation of STAT3 pathway. Its action has been well characterized to affect the conformational structure of STAT3 N-terminal domain (ND)^26^ without affecting the overall expression of the phosphorylated form of STAT3 (pSTAT3). ST3-H2A2 was shown to induce apoptotic death in cancer cells along with robust activation of pro-apoptotic genes^26^. IL10R1-7 (Pal-KLVTLPLISSLQSSE-NH_2,_ all-D) is an analog of the conserved C-terminal motif of IL10R1 (residues 565–578), a region that is critical for the function of the receptor. IL10R1-7 inhibits IL10-dependent growth of MC/9 cells with IC_50_ = 100 nM. IL10R1-14 (Ac-EETQLSPKTSGFGSDTSGHLK–Pal) is an all-D analog of a conserved N-terminal region (residues 313–331) of the cytoplasmic portion of IL10R that does not inhibit growth of MC/9 cells and was used as a negative control. Here we tested peptide inhibitors ST3-H2A2 and IL-10R1-7 for their capacity to deactivate the IL-10-STAT3 pathway *in vivo* and modulate IL-10 downstream activities during a chronic pulmonary infection with Mtb.

We speculated that as a therapy for TB, aerosol delivery of HDT could provide a high concentration of the host-directed drugs at the site of the infection and tubercle lesion and thereby minimize systemic toxicity. We previously developed a procedure applicable to the murine TB model termed “intrapulmonary aerosol delivery”^27–32^. This procedure is not invasive, easy for experienced personnel to administer and allows for repeated administrations of immune modulators (host-directed drugs) or antibiotics in an aerosol form^27,28,30–33^. We also hypothesized that repeated intrapulmonary aerosol delivery of peptide inhibitors ST3-H2A2 and IL10R1-7 to mice chronically infected with *Mtb* should result in decreased pulmonary bacterial load by enhancing the host bactericidal capacity to eliminate the *Mtb* bacilli in the lungs. Furthermore, because STAT3 participates in apoptosis-autophagy pathways, treatment with inhibitors of the IL-10-STAT3 pathway could additionally modulate important checkpoints of the apoptotic and autophagy pathways. The use of lipopeptide inhibitors delivered by aerosol as HDT for TB has not been reported previously. Thus, as a proof of concept, in this study we show that even in the absence of antibiotic TB therapy, it is possible to significantly reduce the pulmonary Mtb bacillary load *in vivo* via modulation of the lung immune environment of the chronically Mtb-infected mice using aerosols of lipopeptide inhibitors.

## Materials and Methods

### Ethics statement

The Institutional Animal Care and Use Committee of Colorado State University approved all animal studies. Studies were performed in accordance with recommendations of the Guide for the Care and Use of Laboratory Animals of the National Institutes of Health.

### Mice and Experimental infection

Six to eight weeks old C57BL/6 female mice were purchased from Jackson (Bar Harbor. ME). The mice were kept in sterile condition in BSL3 facilities and they were rested for a week prior to infection. The mice were then infected with a low dose aerosol infection using the Glass-Col System to deliver ~50–100 *Mtb* (Erdman strain, TMC107; ATCC 35801) bacilli per mouse. Mice were rested during eight weeks until they were randomly assigned to study groups and used to test the therapy under study here.

### Bacterial load determination

Following euthanasia, mouse tissues (lung and spleen) were homogenized using the Next Advance Bullet Blender (Averill Park, NY). Briefly, the left lobe of the lung or spleen were placed in a 1.5 ml sterile, safe lock Eppendorf tubes containing 0.5 ml of sterile saline and 3 × 3.2 mm, sterile stainless steel beads, thereafter the tubes were placed in the Bullet Blender and homogenized during 4 min and 8000 rpm. The bacterial load was determined using serial dilutions of homogenized organs that were plated on 7H11 agar plates and the colony forming units (CFU) in each sample were determined after 3 weeks of incubation at 37 °C. Bacterial load in each animal and organ was expressed as the log_10_ of CFUs.

### Intrapulmonary aerosol delivery of peptide inhibitors

Mice were treated with ST3-H2A2 (n = 5), IL10R1-7 (n = 5) and IL10R1-14 (n = 4) (negative control) peptide inhibitors as follows. Mice were first anesthetized using isofluorane and then received the peptide inhibitors by intrapulmonary aerosol delivery using a MicroSprayer device (MicroSprayer, model IA-C; Penn Century, Philadelphia, PA) attached to an FMJ-250 high pressure-syringe device (Penn Century) as described previously^27,28,30–33^. During the procedure the animals were monitored for regular breathing and clinical symptoms. The mice received six doses during two weeks of 100 µg/50 μl [4 mg/Kg] of 0.9% saline (low endotoxin) per mouse/dose (Experiment 1) or 50 µg/50 µl [2 mg/Kg] of 0.9% saline (low endotoxin) per mouse/dose (Experiment 2) of ST3-H2A2, IL10R1-7 and IL10R1-14 peptide inhibitors via intrapulmonary aerosol delivery.

### ELISAs

Supernatants of centrifuged lung homogenates were screened for pSTAT3 (tyrosine 705, pY705) by ELISA following manufacturer’s recommendations (Life Technologies). The same samples were screened for mouse Bcl-2 (B-cell leukemia/lymphoma 2) and mouse autophagy protein 5 (ATG 5) using ELISA kits from BioAspect (Toronto, Canada) as recommended by the manufacturer.

### Real Time Reverse Transcription PCR

The upper right lobe of the lungs from each mouse were homogenized in Trizol (Invitrogen, Eugene, OR) using the Next Advance bullet blender (Averill Park, NY) and frozen at −80 °C immediately. Real-time PCR was performed using cDNA and Platinum SYBR Green qPCR SuperMix-UDG (Invitrogen) in iQ5 thermo cycler (Bio-Rad, Hercules, CA) to evaluate relative mRNA expression of *il-10, stat3, nos-2, arg-1*. 18S rRNA was used to normalize the expression levels. Primer sequences used were: *il-10* forward primer 5′GCTCTTACTGACTGGCATGAG3′ and reverse primer 5′CAA TACCATTGACCTGCCGAT3′; *stat3* forward primer 5′AATACCATTGACCT GCCGAT3′ and reverse primer 5′AGCGACTCAAACTGCCCT3′; *nos-2* forward primer 5′-GTTCTCAGCCCAACAATACAAGA-3′ and reverse primer 5′GTGGACGGGTCGATGTCAC-3′; *arg-1* 5′CAGAAGAATGGAAGAGTCAG-3′ and reverse primer 5′CAGATATGCAGGGAGTCACC 3′; 18S forward primer 5′GTAACCCGTTGAACCCCATT and the reverse primer 5′ CCATCCAATCGGTAGTAGCG3′. The *18s* rRNA was used as housekeeping gene. The fold induction of *each* transcripts in RNA from lung samples was measured as fold induction of the transcript in samples from *Mtb* infected mice relative to the level of expression of the same transcripts in samples obtained from the lungs of naïve mice.

### Histopathology analysis

The middle right lobe of the lungs of each mouse was placed into a histology cassette and fixed in 4% paraformaldehyde. Samples were inactivated in 4% paraformaldehyde solution for 48 hrs and then processed using standard histological protocols for sectioning and staining with Haematoxylin-Eosin (H&E). Quantitative image analysis of inflammation using H&E staining was performed using the Aperio Imagescope software from Leica Biosystems. Each slide was scanned and then the surface area of each granuloma lesion in each lobule was determined using the Aperio Imagescope software. The mean and SEM score of surface area expressed in μm^2^ for each granuloma was represented.

### Immunohistochemistry

Paraffin embedded blocks from each group of mice were cut in sections of 5–7 μm. Primary antibody raised against murine pSTAT3 (mouse monoclonal IgG1 sc-81523 from Santa Cruz Biotechnology) or IL-10 (Purified anti-mouse IL-10, eBioscience) antigens were used to reveal expression of each antigen. When appropriate cell permeabilization was achieved by incubation with ice-cold methanol. Visualization of the antigen-antibody reaction was done using ImmPACT^tm^ DAB or AEC peroxidase substrate (Vector Lab) as per manufacturer’s instructions.

Negative controls for IHC staining were performed by omitting the primary antibody. In some instances after the ImmPACT DAB incubation, the slides were processed for acid fast staining using BD TB carbofuchsin KF during 30 minutes at room temperature followed by acid-alcohol washes. Finally the slides were counterstained using haematoxylin 560 (SURGIPAD, Leica microsystem) and mounted for microscopic observation using Super Mount mounting media (Biogenex). Sections were examined using an Olympus X70 microscope.

### Cytometric Bead Array Analysis

Supernatants of centrifuged lung homogenates were analyzed using the Cytometric Bead Array (CBA) Kit from BD Biosciences which analyzed IL-6, IL-10, IL-12p70, TNF-α, IFNγ, and MCP-1. Samples were fixed with 4% PFA for at least 24 hrs prior to analysis. A FACsCanto (BD Biosciences and CBA software was used to analyze the samples. Cytokine levels in each sample were calculated by extrapolating the mean fluorescence intensity (MFI) for each sample into the standard curves for every cytokine.

### Antimicrobial products

The quantification of end effector molecules and enzymatic activity involved in the host microbicidal activity in the lungs of mice were performed using supernatants of centrifuged lung homogenates as indicated above. Commercial kits were used to determine the concentration of nitric oxide assay (NO) using the Griess reaction (G 7921, Molecular probes, Invitrogen, Eugene, OR), arginase activity (Bioassay Systems, DARG-200), NADPH oxidase activity (Bioassay Systems, ECNP-100), and lysozyme (Molecular Probes E-22013) activity assays. All assays were performed as per manufacturer’s recommendations.

### Statistical analysis

The data are expressed as the mean ± SEM values from triplicate samples. One-way analysis of variance and the Bonferroni’s Multiple Comparison Test was used for analyzing the p value by comparing all the groups to each other at the confidence interval of 95%. Calculations were performed using Graphpad Prism version 6.00 for Windows (San Diego California USA). P-values < 0.05 were considered significant. In the graphs *p < 0.05; **p < 0.01; ***p < 0.001.

## Results

### Expression of STAT3 and IL-10 in the lungs of mice chronically infected with Mtb

**Figure 1:**
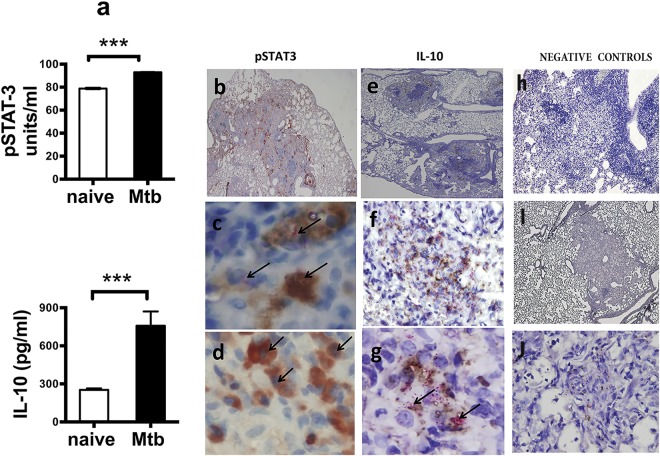
Elevated expression of pSTAT3 and IL-10 in the lungs of mice naïve and chronically infected with Mtb. (**a**) Lung homogenates obtained from naïve (white bars) or chronically Mtb infected (black bars) C57BL/6 mice (n = 5) were assayed by ELISA for pSTAT3 or by CBA for IL-10 to compare the levels of expression between groups of mice. Data represent mean ± SEM where ***p < 0.001. (**b**–**j**) Are photographs of lung tissue sections obtained from mice at 60 days of Mtb infection and stained by IHC only (**b**,**d**,**h**,**i**) or by IHC and acid fast staining (**e**,**c**,**f**,**g**,**j**). (**b**) pSTAT3 (red) positive staining in lesions, perivascular and bronchiolar cellular cuffs (original magnification 4×). (**c**) Co-localization of acid fast positive staining (fuchsia color with arrows) with positive staining for pSTAT3 (brown) in macrophage cells within the granuloma lesion (original magnification100×). (**d**) Positive staining for pSTAT3 (brown) in nuclei of macrophage cells within the granuloma lesion (original magnification 100×). (**e**) IL-10 (brown) within lesions of granulomas (original magnification 4×). (**f**,**g**) Co-localization of acid fast positive staining (fuchsia color) with positive staining for IL-10 (brown) in macrophages within the granuloma lesion (**f**) original magnification 40× and (**g**) original magnification 100×. (**h**) Negative control for pSTAT3 staining in which the first antibody was omitted (original magnification 10×) (**i**,**j**) negative control for IL-10 staining in which first antibody was omitted [I and J original magnification 10× and 100× respectively].

Up-regulation of expression of IL-10 in the lungs of chronically Mtb infected mice is well established^12–14,30,34,35^ but the expression of STAT3 or its phosphorylated form, pSTAT3, have not been previously examined. Thus, we first assessed the expression of STAT3 and IL-10 in the lungs of naïve mice and mice with a chronic infection of Mtb using ELISA, CBA (Fig. 1a) and immunohistochemistry (IHC) **(**Fig. 1b–j**)**. The results demonstrated that lung homogenates obtained from mice following 60 days of infection (Fig. 1a, black bars) have increased levels of pSTAT3 and IL-10 proteins when compared to similar samples obtained from naïve mice. When lung tissue sections from mice with chronic infection of Mtb were analyzed by IHC (Fig. 1b–j), many macrophages and occasional foamy cells and lymphocytes (Fig. 1b,c) located within the granulomatous lesion were positive for expression of pSTAT3. pSTAT3 IHC followed by acid-fast bacilli (AFB) staining identified cells within the granulomatous lesions in which the pSTAT3 (brown) and AFB (fuchsia) color co-localized within the same cell (Fig. 1c). pSTAT3 staining was observed in cytosol and nuclei of cells (Fig. 1c,d). Similar tissue areas were also positive for IL-10 in macrophage and lymphocytes within the granuloma (Fig. 1e–g). Likewise, when staining for IL-10 by IHC was followed by AFB staining to visualize the Mtb bacilli within the lesions, it was revealed that IL-10 and AFB are located within the same cells (Fig. 1f,g) but unlike staining for pSTAT3, IL-10 staining was not seen in the nuclei. IHC specific staining in the lung sections was demonstrated by omitting the primary antibody for pSTAT3 **(**Fig. 1h**)** and IL-10 **(**Fig 1I,j**)**. Overall these results demonstrated that pSTAT3 and IL-10 are upregulated in cells located at the site of the lesions in the lungs of mice chronically infected with Mtb. Furthermore this analysis showed that some cells at the site of the lesions express high levels of pSTAT3 and IL-10 and co-localize with AFB positive staining.

### Pulmonary bacterial load after local pulmonary HDT with peptide inhibitors

**Figure 2:**
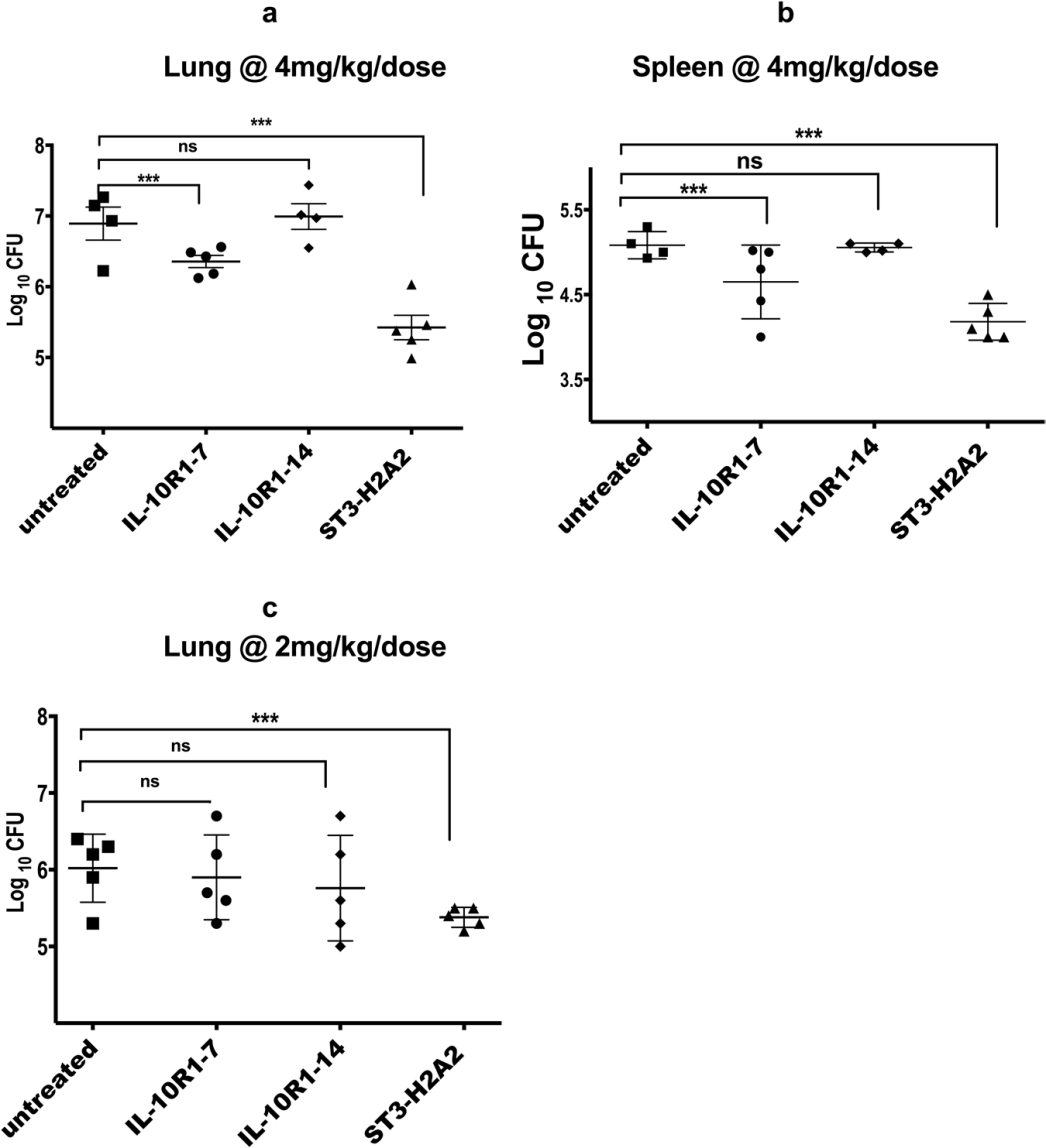
Pulmonary bacterial load after local pulmonary HDT with peptide inhibitors IL10R1-7, IL10R1-14 and ST3-H2A2. Mice were infected with a low dose aerosol of *Mycobacterium tuberculosis [Erdman]* strain. Sixty days after the infection, mice were randomly assigned to groups. Each group of mice were either not treated (n = 4) or treated three times a week for two weeks via local intrapulmonary aerosol HDT with the peptides IL10R1-7 (n = 5), IL10R1-14 (n = 4) and ST3-H2A2 (n = 5). Twenty-four hours after the last dose, mice were euthanized and the lungs and spleens were harvested and prepared for bacterial load determination. (**a**,**c**) The log_10_ of colony forming units (CFU) obtained from lung (**a**,**c**) or spleen (**b**) samples of each mouse in the group of mice not receiving treatment or mice treated with (**a**,**b**) 4 mg/Kg/dose or (**c**) 2 mg/Kg/dose of peptide inhibitors with IL10R1-7 (n = 5), IL10R1-14 (n = 5) and ST3-H2A2 (n = 5) are shown ***p < 0.001; ns = no statistical significance.

We subsequently determined the effect of pharmacological intervention targeting the IL-10-STAT3 signaling pathway on the pulmonary (Fig. 2a,c) and splenic Mtb bacterial load (Fig. 2b). The results demonstrated that the pulmonary bacterial load for Mtb challenged mice treated with 6 doses of 100 μg/dose (4 mg/Kg) of ST3-H2A2 during two weeks period was reduced by 1.7 log_10_ CFU when compared to similarly infected control groups. Treatment of chronically *Mtb* infected mice with the peptide inhibitor IL10R1-7 also decreased the pulmonary CFU by 0.6 log_10_ whereas similar treatments with the peptide inhibitor IL10R1-14 used as a negative control showed not statistical significance in the pulmonary CFU when compared to untreated control groups (Fig. 2a). Likewise, the splenic bacilli load was reduced by 0.9 and 0.4 log_10_CFU in mice treated with ST3-H2A2 and IL-10R1-7 respectively (Fig. 2b). In a separate experiment when chronically Mtb infected mice were treated with a lower 2 mg/Kg dose of ST3-H2A2, IL10R1-7 or IL10R1-14 only the group of mice receiving ST3-H2A2 had reduced pulmonary CFU (by 0.6 log_10_) when compared to control mice **(**Fig. 2c**)**. No changes in the bacterial load of the spleen were observed with this therapy. Most importantly, the possibility that the lipopeptides under investigation have anti-microbial activity by themselves was discarded in a minimum inhibitory assays (MIC) assay. This assay found that the lipopeptide inhibitors lacked any biologically relevant MIC activity versus either *Mtb* H37Rv or Erdman strains when tested up to 16 mg/L in two different microbiological media. All together these results show that intermittent administration of aerosols containing peptide inhibitors ST3-H2A2 and IL10R1-7 to the chronically Mtb infected lung can effectively reduce the bacilli load.

### Effect of pulmonary treatment with peptide inhibitors on *stat3* and *Il-10* transcripts and protein

**Figure 3:**
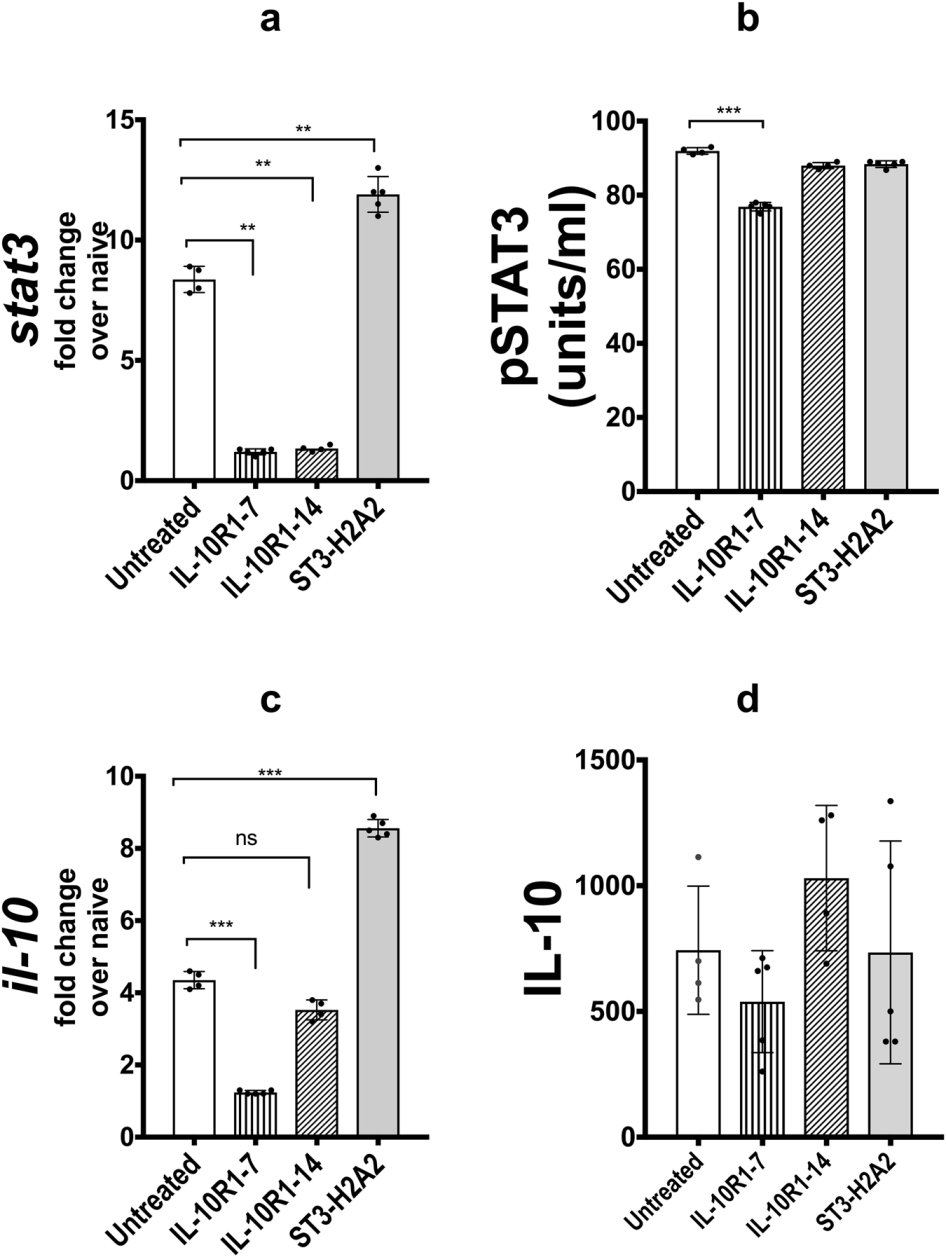
Effect of local pulmonary HDT with IL10R1-7, IL10R1-14 and ST3-H2A2 peptide inhibitors on the *stat3* and *Il-10* transcripts and protein levels. (**a**,**c**) Lung samples from mice as in Fig. 2 were analyzed by RT-PCR for mRNA transcript expression of *stat3* and *il10* using lung homogenates based on the expression of 18S. Briefly, the lungs were harvested and samples were collected in Trizol for RNA extraction and qRT-PCR assay was performed to quantify *stat3* or il-10 mRNA. (**b**,**d**) - Lung homogenates as in Fig. 2 were assayed by ELISA for pSTAT3 or by CBA for IL-10 cytokine. Peptide inhibitor treatment regimens are indicated as: No treatment, IL10R1-7, IL10R1-14 and ST3-H2A2 treatment. Data represent mean ± SEM where *p < 0.05; **p < 0.01; ***p < 0.001 and ns; no statistical significance.

qRT-PCR was used to determine the effect of local pulmonary HDT using ST3-H2A2, IL10R1-7 and IL10R1-14 on the expression of *stat3 and Il-10* transcripts (Fig. 3a,c). The data shows significant increase in the levels of pulmonary expression of *stat3* and *il-10* transcripts mRNA after treatment with ST3-H2A2. The levels of pSTAT3 and IL-10 in the lung homogenates obtained from the same mice were analyzed via ELISA and CBA respectively **(**Fig. 3b,d**)**. ELISA revealed little change in the levels of pSTAT3 or IL-10 expression in lung samples after two weeks of treatment with ST3-H2A2 when compared to control mice chronically infected with Mtb. On the other hand, samples from mice treated with IL10R1-7 presented lower levels of the pSTAT3 and STAT3 than those from control mice when analyzed by either RT-PCR or ELISA. Samples from mice receiving similar treatment with the IL10R1-14 peptide inhibitors showed low or no significant changes in the expression of pSTAT3 and IL-10 but had significant reduction for *stat3* mRNA when compared to control uninfected mice. Thus the effect in the pulmonary expression of IL-10 and STAT3 for each of the peptide inhibitors after aerosol administration to the lungs differs considerably.

### Effect on the expression of antimicrobial effector molecules after local intrapulmonary aerosol HDT with peptide inhibitors

**Figure 4:**
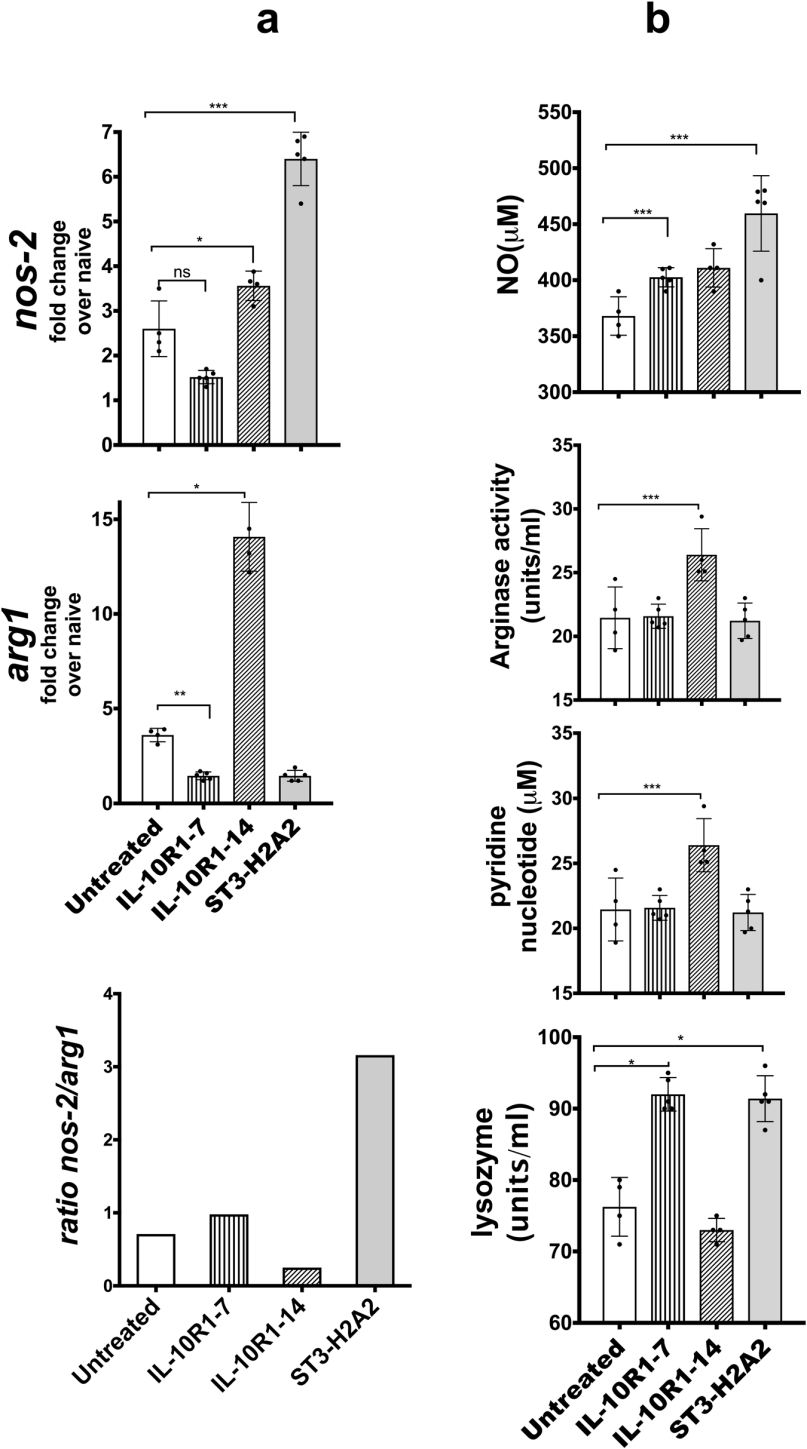
Effect on the expression of antimicrobial effector molecules after local intrapulmonary aerosol HDT with peptide inhibitors. (**a**) RNA samples as in Fig. 3 were also assayed by real-time RT-PCR to determine expression of *nos2* and *arg1*. (**b**) Lung homogenates from each mouse as in Fig. 2 were used to determine the activity of the NOS-2 by the Griess reaction. Arginase, lysozyme and NADPH activities were also measured as described in Material and Methods. Peptide inhibitor treatment regimens are indicated as: No treatment, IL10R1-7, IL10R1-14 and ST3-H2A2 treatment. Data represent mean ± SEM where *p < 0.05; **p < 0.01; ***p < 0.001.

We predicted that by interfering with the IL-10 and STAT3 signaling pathway, antibacterial mechanisms would be induced because previous studies indicated that persistent activation of the IL-10-STAT3 pathway inhibits the antimicrobial capacity of the cells^8,15,36^. The NOS-2 and Arg-1 enzymes are critical to the intricate regulation of bactericidal activity [mediated by NOS-2 and NADPH] versus tissue repair activities [mediated by Arg-1] ascribed to macrophages^7,8,36^. Thus, we monitored the outcome of the pulmonary aerosol HDT with peptide inhibitors by analyzing the levels of *nos-2* and *arg1* mRNAs via qRT-PCR as well as the enzymatic activity of NOS2, Arginase, NADPH oxidase and lysozyme in lung homogenates obtained from each mouse included in this study (Fig. 4). The expression of *nos2* mRNA increased significantly in lung samples obtained from mice treated with the peptide inhibitor ST3-H2A2 (Fig. 4a) when compared to similar samples obtained from control untreated mice chronically infected with Mtb without changes in expression of the *arg1* mRNA in the same samples. Samples from mice treated with the IL10R1-14 demonstrated a 2- to 10-fold increase in both *nos2* and *arg1* transcript expression respectively when compared to untreated control mice (Fig. 4a). Finally, samples obtained from the group of mice treated with IL10R1-7 showed reduction in transcript expression for *arg1* but no significant change in *nos2* (Fig. 4a). Interestingly, the total ratio of *nos-2/arg1* (Fig. 4a) appeared notably increased in mice treated with the peptide inhibitor ST3-H2A2 and decreased in IL-10R1-14 when compared to untreated control mice.

The enzymatic activity of NOS-2, Arginase, NADPH oxidase and lysozyme were also evaluated in lung homogenates using commercial kits. The activity for each of these enzymes was inferred by measuring concentrations of NO [NOS2], DQ substrate [lysozyme] and pyridine nucleotide [NADPH] in lung samples. NO and lysozyme activities were significantly elevated in mice treated with ST3-H2A2 or IL10R1-7 (Fig. 4b) whereas the activity of Arginase was reduced or not changed when compared to similar samples obtained from control untreated mice chronically infected with Mtb. Similar samples obtained from IL10R1-14 showed lower or no increase for NOS2 and lysozyme activity but had increased arginase activity when compared to control untreated mice. Thus, the effect in the pulmonary expression of end products needed for antimicrobial activity in the lungs was enhanced to a greater extent in the lungs of mice receiving ST3-H2A2 and to a lesser extend in mice receiving IL10R1-7 or IL10R-1-14.

### Changes in the profile of Th1 cytokines after local intrapulmonary aerosol HDT with peptide inhibitors

**Figure 5:**
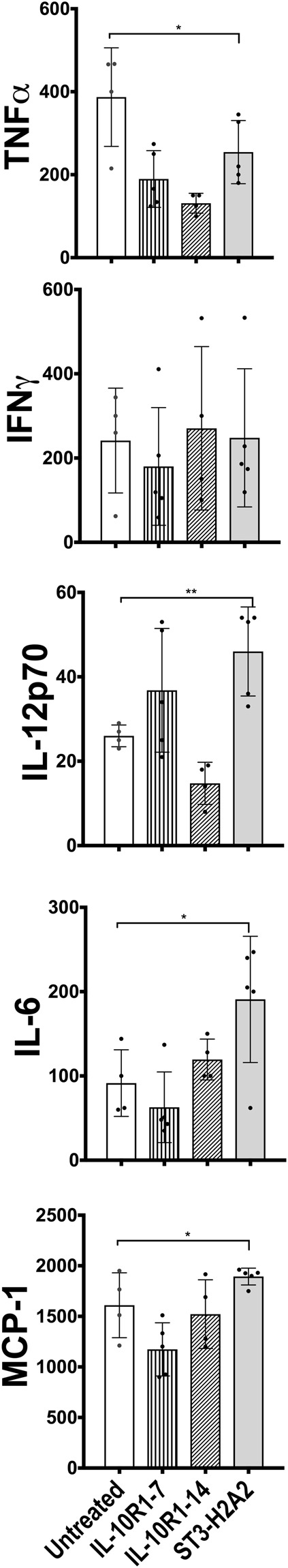
Changes in the profile of Th1 cytokines after local intrapulmonary aerosol HDT with peptide inhibitors. Cytometric bead assay [CBA] analyzing IL-6, IL-10, IL-12p70, TNF-α, IFNγ, and MCP-1 was used to determine if local intrapulmonary aerosol HDT with peptide inhibitors ST3-H2A2, IL10R1-7 and IL10R1-14 results in changes on the levels of inflammatory cytokines. The concentration of each analyte was determined by extrapolating the mean fluorescence intensity for each sample into the standard curves for every cytokine. Data is expressed as pg/ml of sample. Peptide inhibitor treatment regimens are indicated as: No treatment, IL10R1-7, IL10R1-14 and ST3-H2A2 treatment. Data represent mean ± SEM of triplicates where *p < 0.05; **p < 0.01.

The Th1 responses and regulation of the recruitment of Th1 immune cells to the site of infection are characterized by expression of key cytokines and chemokine such as TNFα, IL-12, IFNγ, IL-6, IL-10 and MCP-1 among others. Cytometric bead assays revealed that local intrapulmonary aerosol HDT with ST3-H2A2 and IL10R1-7 has significant impact on the expression of these cytokines (Fig. 5). The levels of the Th1 cytokine and highly inflammatory cytokine TNFα were reduced after treatment with all three peptides but surprisingly the levels of IFNγ expression in the lungs of mice treated with local HDT of peptide inhibitors ST3-H2A2 and IL10R1-7 were also decreased or unchanged when compared to control mice. Interestingly, the expression levels for the cytokines IL-12p70 and IL-6 were increased in the lungs of mice treated with local HDT with ST3-H2A2 but decreased in similar samples obtained from the IL10R1-7 treated mice and when compared to control mice. Overall, these results indicated that the Th1 cytokines were still expressed in the lungs after aerosol administration of peptide inhibitors and there was not an overridden increase in the levels of expression of highly inflammatory cytokines such as TNFα or MCP-1.

### Effect of the local HDT with peptide inhibitors on the lung histology

**Figure 6:**
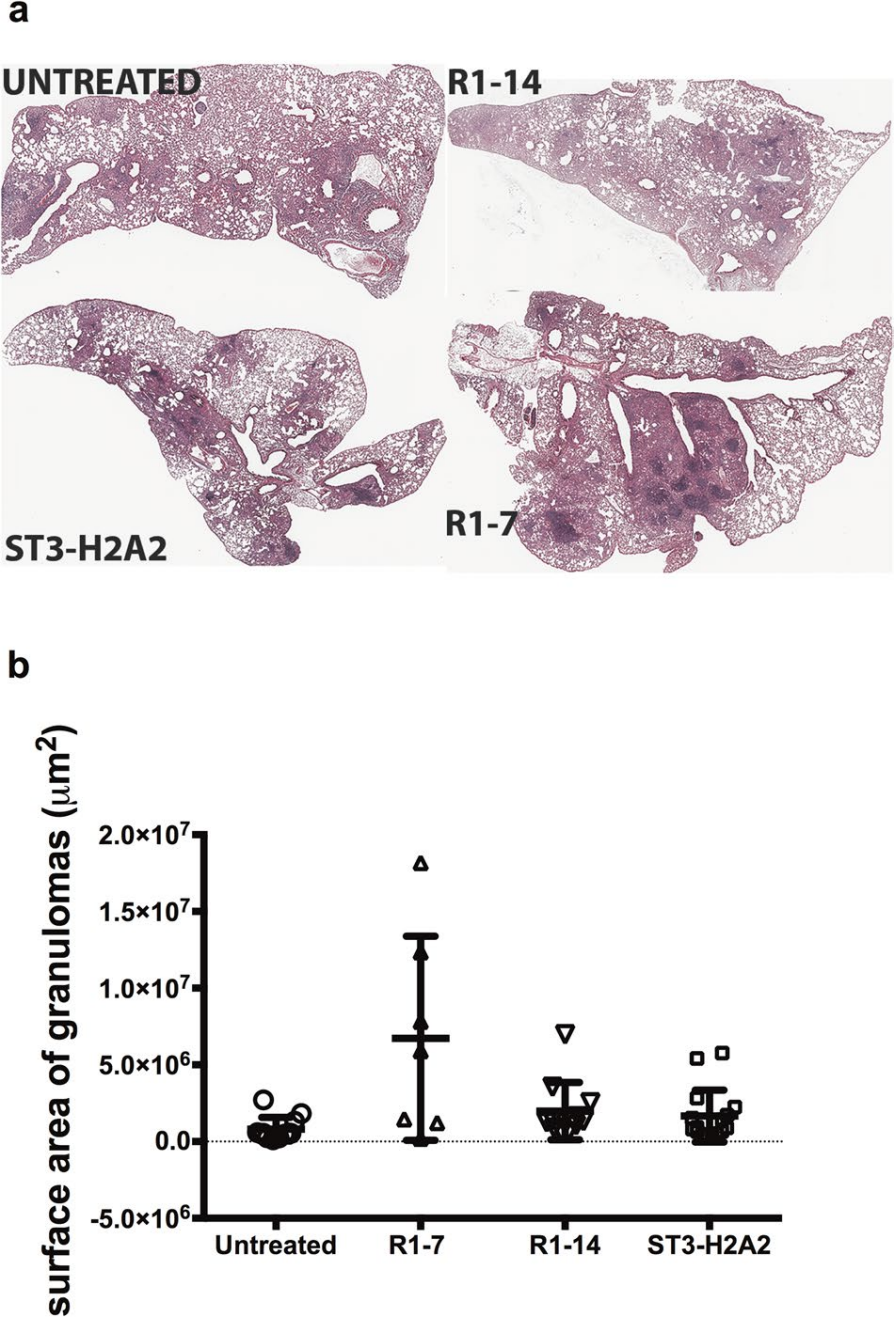
Effect of the local HDT with peptide inhibitors in the lung histology. The accessory lobe of the lungs of each mouse was placed into a histology cassette and these samples were processed for H&E staining. (**a**) Representative images from one of the lung lobules from one mouse obtained from chronically Mtb infected mice receiving no therapy [Mtb untreated; upper left photo] or similar mice receiving therapy with IL10R1-14 [upper right photo], ST3-H2A2 [lower left photo] or IL10R1-7 [lower right photo] peptide inhibitors. All images are shown at original magnification ×4 after scanning of the H&E stained sections (**b**) Quantitative image analysis of area of inflammation using H&E staining. The graph represents the scattered plot of the area in μm^2^ (and mean value) for each granuloma appearing in each of the lung lobule sections from each group of mice under study.

The outcome of the pulmonary administration of HDT via aerosol of peptide inhibitors was also analyzed at the histological level. Lung tissue sections from each group of mice were stained by H&E (Fig. 6a). All the groups presented granuloma lesions in the lungs. Accumulations of macrophages, many foamy cells and clusters of lymphocytes formed the granulomas. The most noticeable observation when comparing the histopathology between H&E stained lungs sections from all groups of mice was increased numbers of clusters and highly packed lymphocytes in lung tissue sections obtained from groups of mice treated with either ST3-H2A2 or IL10R1-7 and when compared to tissue sections obtained from Mtb infected mice treated with IL10R1-14 or a control group (Fig. 6a). The graph in Fig. 6b represents the scattered data of the area for each granuloma appearing in each of the lung sections from each group of mice under study. Thus the accessory lobe of the lungs of each mouse was placed into a histology cassette, these samples were processed for H&E staining and the average of the surface area of all granuloma lesions appearing in H&E slides was measured as indicated in the methods section (Fig. 6b). The results indicated that only mice receiving the peptide inhibitor IL-10R1-7 had increased the average area of the granulomas. These observations indicated that administration of aerosols containing peptide inhibitors targeting the IL-10-STAT3 pathway had a noticeable effect in the inflammatory response in the lungs of mice treated with the peptide inhibitor IL-10R1-7 but this effect was minimal in mice treated with ST3-H2A2 and IL-10R1-14.

### Changes in important checkpoints of the apoptosis and autophagy pathways after local HDT with peptide inhibitors

**Figure 7:**
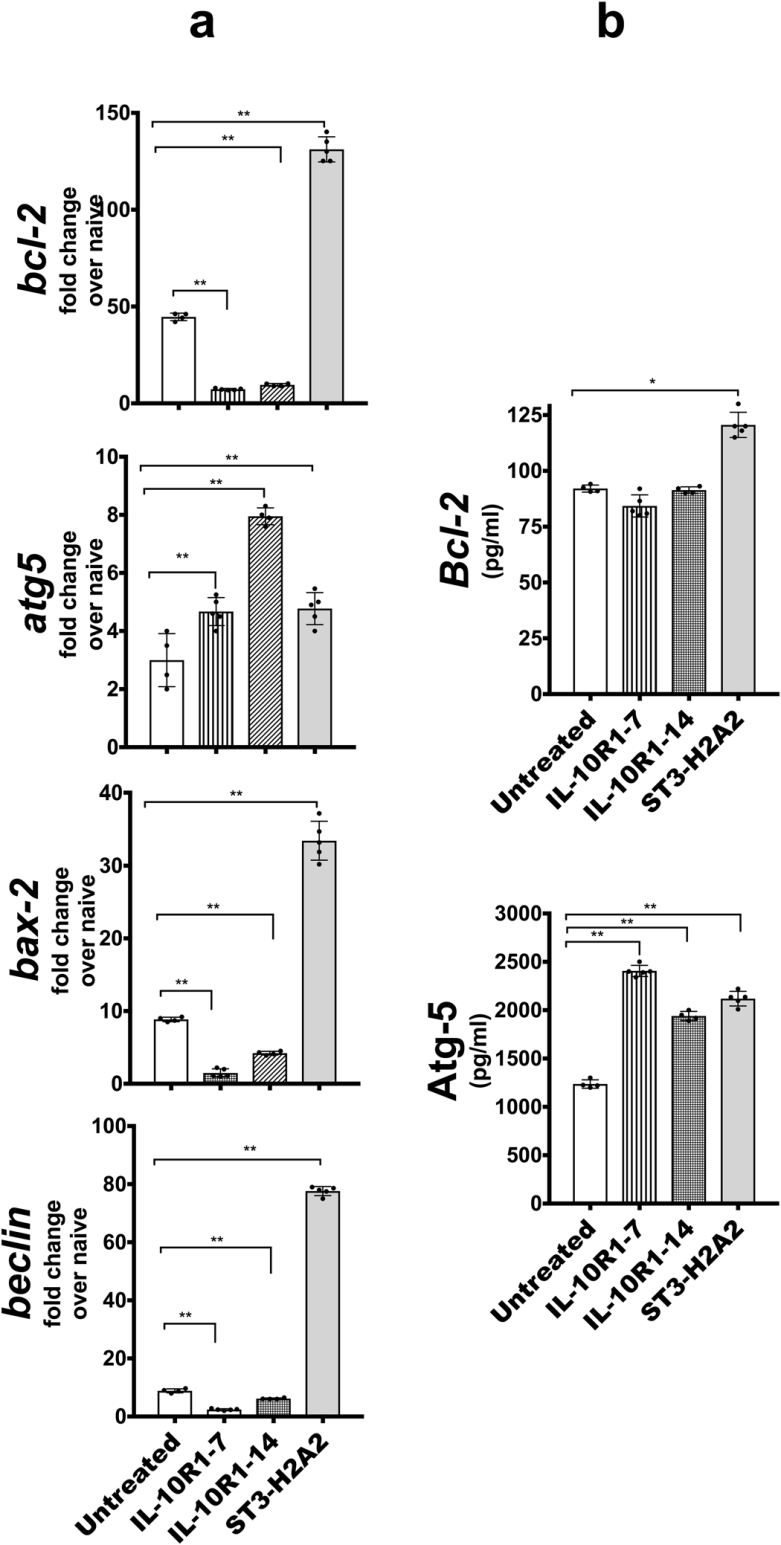
Changes in expression of important checkpoints of apoptosis and autophagy pathways after local HDT with peptide inhibitors. (**a**) Lung samples from mice as in Fig. 2 were analyzed by RT-PCR for mRNA transcript expression of *bcl-2, bax, atg5* and *beclin-1* using lung homogenates of C57BL/6 mice based on the expression of 18 S. Briefly, the lungs were harvested and samples were collected in Trizol for RNA extraction and RT-PCR assay. (**b**) Lung homogenates as in Fig. 2 were assayed by ELISA for Bcl-2 and Atg-5. Peptide inhibitor treatment regimens are indicated as: No treatment, IL10R1-7, IL10R1-14 and ST3-H2A2 treatment. Data represent mean ± SEM of triplicates where *p < 0.05; **p < 0.01; ***p < 0.001.

We hypothesized that localized HDT via administration of peptide inhibitors targeting the IL-10-STAT3 pathway should modulate the expression of important checkpoints in the apoptotic and autophagy pathways. To test this hypothesis, we used qRT-PCR and ELISA to analyze transcripts or lung homogenate samples respectively obtained from each of the mice utilized in this study (Fig. 7a,b). qRT-PCR showed more than 3-fold increase in expression of all transcripts under study here; the *bcl-2, bax, atg5* and *beclin-1* in the samples obtained from mice receiving ST3-H2A2, whereas samples obtained from the IL10R1-7 and IL10R1-14 treated mice had increased expression of *atg5* transcript only but had reduced expression of the *bcl-2, bax and beclin-1* transcripts. When lung homogenates from each mouse were used to determine the levels of expression of Bcl-2 and Atg-5 protein using ELISA, the results were similar to those obtained by qRT-PCR. Thus, Bcl-2 was significantly increased only in samples from ST3-H2A2 treated mice when compared to samples from control untreated mice with a chronic infection with Mtb. Atg-5 was increased in samples obtained from mice treated with all three peptide inhibitors when compared to control mice.

## Discussion

Self-limited host pulmonary bactericidal capacity in patients chronically infected with *Mycobacterium tuberculosis* (Mtb) is without doubt the major barrier to overcoming a chronic infection with this bacillus. The results of this study showed that a chronic infection with Mtb in mice leads to high expression levels of the phosphorylated form of STAT3 (pSTAT3) in the lungs and that pSTAT3 localizes to macrophages and lymphocytes in the granuloma lesions. Likewise, as reported previously^12–14,30,34,35^ mice chronically infected with Mtb also have elevated levels of expression for the immunosuppressive cytokine IL-10. An interesting finding was that some macrophages within granuloma lesions containing acid fast-positive bacteria were also positive for staining for pSTAT3 and IL-10 indicating that some Mtb infected macrophages within the granulomas may undergo persistent activation of the IL-10-STAT3 pathway. Previous studies in the TB^7,36^ and cancer fields^37^ have identified shifts in the STAT3-IL-10 pathway as a cause of reduced bactericidal and tumoricidal capacity of macrophages at the site of the lesion. Consequently, we hypothesized that local and pulmonary pharmacological intervention targeting the STAT3-IL-10 pathway should be capable of reducing the bacterial burden in the lungs of mice chronically infected with Mtb. We tested whether a therapy consisting of an aerosol delivery of peptide inhibitors targeting the N terminal domain (ND) of the STAT3^26^ or the IL-10R1 could change the course of a pulmonary chronic infection with Mtb in mice. As a direct result of this therapy we observed that the pulmonary bacterial burden was reduced in mice treated with either of the peptide inhibitor ST3-H2A2 or IL10R1-7 compared to the bacterial burden of chronically Mtb infected mice not receiving treatment or a similar group of mice treated with the control peptide inhibitor IL10R1-14. Interestingly, in agreement with our studies a previous study also found that interference with STAT3 pathway induced expression of IL-10^38^. Thus, the first conclusion derived from this study is that a short regimen of HDT consisting of intrapulmonary aerosol of peptide inhibitors targeting that STAT3-IL-10 pathway delivered to mice with a chronic infection with Mtb is capable of reducing by more than 95% the pulmonary bacilli load. The second conclusion is that even in the absence of antibiotics against Mtb bacilli, it is possible to reduce the Mtb bacilli burden in the lungs of the chronically infected host.

Interestingly, increased bactericidal effect after ST3-H2A2 treatment was paralleled with increased abundance of *stat3 and il-10* transcripts and IL-6 protein whereas the same decreased after IL-10R1-7 or IL-14R1-14 treatment. ST3-H2A2 is known to be a highly selective inhibitor of STAT3 ND that binds to the N-terminal domain (ND) and inhibits STAT3 signaling without affecting expression of STAT3 and pSTAT3 in cancer cell lines^26^. We concluded that *in vivo* interference with the STAT3 activity may have affected other pathways that lead to overcompensation and upregulation of expression of IL-6 and *il-10* and subsequent increased STAT3 expression yet STAT3 functions appeared to be interfered by ST3-H2A2. Furthermore, the large reduction in the pulmonary bacterial load seen in mice treated with STAT3 N-domain inhibitor correlated with changes in the overall bactericidal capacity of the lungs and most likely this effect was host-mediated as the peptide inhibitors had no effect on Mtb growth in an *in vitro* assay. Mice treated with ST3-H2A2 had increased *nos2/arg1* ratio and increased enzymatic activity of NOS-2 and lysozyme, another enzyme known to have strong bactericidal activity against Mtb^39,40^. Our *in vivo* studies here are in line with previous *in vitro* studies^24^ indicating STAT3 represses NOS in human macrophages upon Mtb infection. Furthermore, mice treated with an analog of a conserved N-terminal region of IL10R1 cytoplasmic domain (residues 313–331) IL10R1-14 did not show changes in the bacterial load. Mice treated with the analog of the conserved C-terminal motif of IL10R1, IL10R1-7 had lower reduction in the bacterial load than ST3-H2A2 treated mice with slight increase in *nos2/arg1* ratio but with a notable increase in lysozyme activity. Likewise, the changes observed for bactericidal activities in the IL10R1-7 treated mice were less pronounced than those observed for mice treated with the STAT3 ND inhibitor, most likely, because of the more significant changes in expression profiles caused by direct modulation of STAT3 transcriptional activity. However, it is also possible that less optimized IL10R1 antagonist has less favorable biodistribution and metabolic stability.

While the desired outcome of a local HDT administered directly to the lungs of mice is reduction in the load of the Mtb bacilli, control of the homeostatic pulmonary inflammatory process during therapy is vital. Staining of lung sections from each mouse by H&E revealed that all groups of mice regardless of their bacterial load still presented granuloma lesions and that mice receiving peptide inhibitor IL-10R1-7 presented high inflammatory response when compared to mice not receiving therapy or mice treated with ST3-H2A2 or IL-10R1-14. It was also noticed that lymphocyte clusters within the granulomas in mice treated with ST3-H2A2 and the IL10R1-7 [both of which had lower bacterial load than other groups of mice] had higher infiltration of lymphocytes than the other groups of mice. Changes in the nature and function of pulmonary lymphocytes during local HDT with these peptide inhibitors remains to be elucidated but the histological evaluation suggests that targeting the IL-10-STAT3 pathway results in changes in the recruitment or proliferation stage of lymphocytes population to the site of the granulomatous lesion as well. In agreement with this observation, a recent study^41^ suggested that deactivation of arginase 1 augments T cell proliferation and activation within the tuberculous granuloma. Histopathological analysis revealed that the expression levels of inflammatory cytokines/chemokines TNFα, IFNγ, and MCP-1 were reduced or remained unchanged in mice treated with peptide inhibitors when compared to chronically Mtb infected mice not receiving the therapy. Previous studies have shown that IFNγ activates expression of the NOS-2^42^ and it would have been expected that higher NOS-2 activity would have correlated with higher levels of expression of IFNγ. However, in these studies, pharmacological intervention in the IL-10-STAT3 pathway resulted in reduced pulmonary bacilli load with lower or not changes in expression of the IFNγ cytokine suggesting that deactivation of the IL-10-STAT3 pathway affects IFNγ function.

Apoptosis and autophagy along with the action of effector molecules produced by NOS-2, NADPH oxidase and lysozyme are also important ways of deploying bactericidal activity. Thus, modulating the apoptotic and autophagy pathway should also be considered during development of new HDT protocols. The IL-10-STAT3 pathway is known to affect the proliferation capacity of cells within tumors and to regulate important checkpoints of cell autophagy and apoptosis^37,43^. In the present studies we observed increased expression at the transcript [Bcl-2, Bax, Beclin-1 and Atg5] and protein [Bcl-2 and Atg5] levels after treatment with the STAT3 ND inhibitor, while only Atg 5 was increased after therapy with IL10R1-7, both consistent with high reduction in the bacterial load and increase pulmonary bactericidal activity. It is unclear whether apoptosis and/or autophagy were indeed responsible for increased bactericidal activity in the lungs of these mice but the fact that the levels of expression for the key proteins participating in these pathways were modified upon *in vivo* therapy open important venues for HDT targeting the apoptosis and autophagy pathways.

A very recent report^21^ demonstrated strong association between constitutive expression of pSTAT3 and high IL-6/IL-10 co-expression in tuberculosis patients^21^ with impaired T-cell function. Interestingly, simultaneously, another publication used genetically modified mice for STAT3 and regulator suppressor of cytokine signaling (SOCS)3 to demonstrate that STAT3 expression in myeloid cells negatively affects control of infection with Mtb by increasing Th17 responses in bacteria-specific CD4 T cells. In these studies, we focused on the interference of IL-10-STAT3 and its effects in global host bactericidal capacity against Mtb but it is also possible that IL-6/SOCS3 are also affected by this therapeutic approach.

In summary, local administration of an aerosol therapy consisting of peptide inhibitors for the IL-10-STAT3 pathway appears to be a promising therapeutic approach for targeting the Mtb bacilli in the lungs. The study also suggests that delivery of immune modulators through aerosols directly into the lungs can change the course of a chronic infection without the danger of a hyper-inflammatory reaction. These studies also pave a path for the development of aerosol HDTs aiming at targeting specific immune checkpoints of the lungs to determine their specific contribution in pulmonary immunity or the disease. All together suggest that HDT targeting STAT3 signaling for TB is a feasible approach and warrant further investigation. Further studies will evaluate activation of STAT3 signaling, mechanism of action and efficacy of this HDT when combined with standard chemotherapy for TB.
